# Dormant tumor cells expressing LOXL2 acquire a stem-like phenotype mediating their transition to proliferative growth

**DOI:** 10.18632/oncotarget.12109

**Published:** 2016-09-19

**Authors:** Keren Weidenfeld, Sagi Schif-Zuck, Hanan Abu-Tayeh, Keunsoo Kang, Ofra Kessler, Marina Weissmann, Gera Neufeld, Dalit Barkan

**Affiliations:** ^1^ Department of Human Biology, University of Haifa, Haifa, Israel; ^2^ Department of Microbiology, Dankook University, Cheonan, Republic of Korea; ^3^ Cancer Research and Vascular Biology Center, The Bruce Rappaport Faculty of Medicine, Technion, Israel Institute of Technology, Haifa, Israel

**Keywords:** dormant tumor cells, breast cancer recurrence, cancer stem cells, epithelial mesenchymal transition, LOXL2

## Abstract

Recurrence of breast cancer disease years after treatment appears to arise from disseminated dormant tumor cells (DTC). The mechanisms underlying the outgrowth of DTC remain largely unknown. Here we demonstrate that dormant MCF-7 cells expressing LOXL2 acquire a cancer stem cell (CSC)-like phenotype, mediating their outgrowth in the 3D BME system that models tumor dormancy and outgrowth. Similarly, MCF-7-LOXL2 cells colonizing the lung transitioned from dormancy to metastatic outgrowth whereas MCF-7 cells remained dormant. Notably, epithelial to mesenchymal transition (EMT) of MCF-7-LOXL2 cells was required for their CSC-like properties and their transition to metastatic outgrowth. These findings were further supported by clinical data demonstrating that increase in LOXL2 mRNA levels correlates with increase in the mRNA levels of EMT and stem cells markers, and is also associated with decrease in relapse free survival of breast cancer patients. Notably, conditional hypoxia induced expression of endogenous LOXL2 in MCF-7 cells promoted EMT and the acquisition of a CSC-like phenotype, while knockdown of LOXL2 inhibited this transition. Overall, our results demonstrate that expression of LOXL2 endowed DTC with CSC-like phenotype driving their transition to metastatic outgrowth and this stem-like phenotype is dependent on EMT that can be driven by the tumor microenvironment.

## INTRODUCTION

Recurrence of breast cancer often follows a long latent period in which there are no signs of cancer, and metastases may not become clinically apparent until many years after removal of the primary tumor and adjuvant therapy. A likely explanation of this phenomenon is that tumor cells which are resistant to conventional therapies have seeded metastatic sites but remain dormant for long periods of time [1–5].

The existence of dormant (quiescent) tumor cells (DTC) at secondary sites has been previously described. Several lines of evidence suggest that DTC may reside as quiescent solitary cells in the bone marrow, lymph nodes, and blood circulation of breast cancer patients [6–8]. Moreover, recent studies demonstrate that dissemination of tumor cells may occur at an early stage of tumor progression [1, 4, 9, 10]. Little is known about the molecular mechanisms by which DTC emerges from dormancy (quiescence) to metastatic growth [5]. Thus, it is imperative to study the mechanisms underlying this switch.

We recently demonstrated that a permissive tumor microenvironment is required for the switch of DTC to metastatic growth [11–13]. In particular the fibrotic microenvironment and remodeling of the extracellular matrix (ECM) were part of the tumor permissive microenvironment, supporting the emergence from tumor dormancy to metastatic growth. Furthermore, lysyl oxidase (LOX) family members, (a family of matrix remodeling enzymes), were shown to play an important role in establishing such a permissive microenvironment for metastasis to occur [14–16].

Lysyl Oxidase Like 2 (LOXL2), a member of the LOX family, facilitates ECM remodeling [14], induces epithelial to mesenchymal transition (EMT) [17, 18] and has been shown to be hypoxia-regulated in fibroblasts and renal tubular epithelial cells [19], and plays an important role in generation of the pathologic stroma that constitutes the tumor microenvironment, ultimately leading to a more aggressive disease [14, 20]. Accordingly, up-regulation of LOXL2 has been observed in breast cancer where high levels of LOXL2 have been associated with more aggressive phenotype and metastatic potential [18, 20–23]. Moreover, LOXL2 expression has been demonstrated to clinically correlate with metastasis and decreased survival of breast cancer patients [22]. Hence, based on our previous finding and emerging experimental evidence on the role of LOXL2 as a modulator of the metastatic niche [14, 15, 24], we hypothesized that LOXL2 expression in DTC will facilitate their emergence from tumor dormancy to metastatic growth by inducing cellular changes such as EMT and acquiring stem-like properties. We have used MCF-7, a luminal breast cancer cell line, cultured in a model of three-dimensional growth factor reduced basement membrane extract (3D BME). We have previously demonstrated that this model system can recapitulate quiescent dormancy of these cells as displayed *in vivo* [13, 25]. Here we demonstrate for the first time that expression of LOXL2 in DTC can promote their acquisition of a CSC-like phenotype and promote their transition to metastatic outgrowth.

## RESULTS

### LOXL2 expression in dormant MCF-7 cells promotes their EMT in the 3D BME system

**Figure 1:**
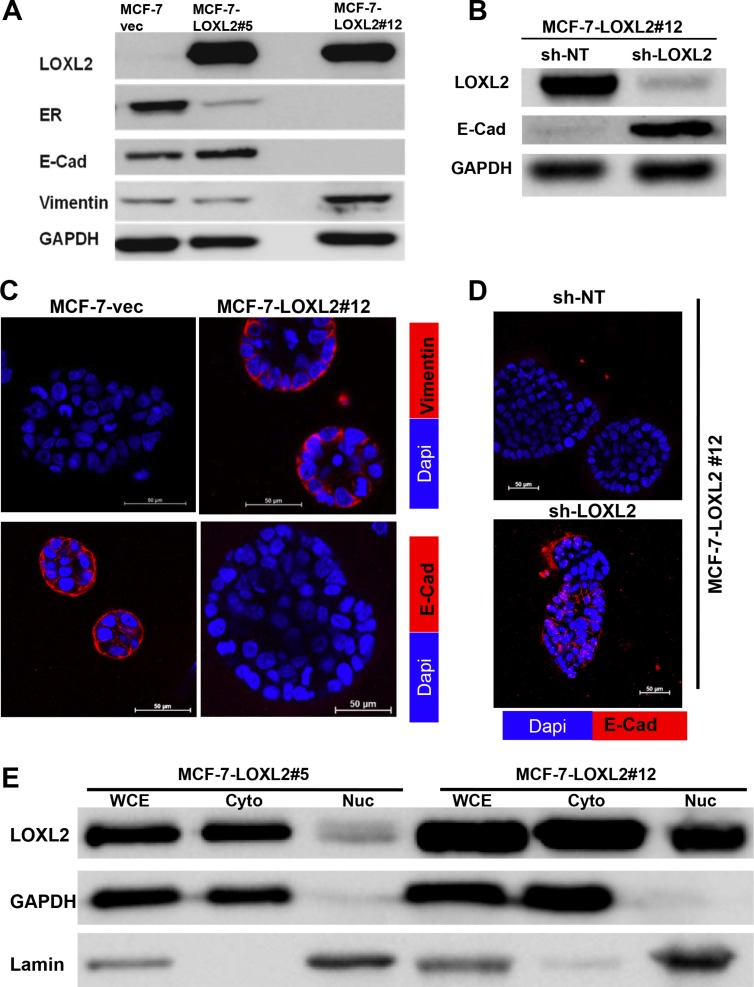
Characterization of MCF-7-LOXL2 cell lines for EMT and expression of luminal markers (**A**–**B**) Western-blot analysis of MCF-7-LOXL2 clones (MCF-7-LOXL2#12, MCF-7-LOXL2#5) and of MCF-7-LOXL2#12 cells stably expressing either sh-non-target (sh-NT) or sh-LOXL2 (sh-LOXL2) for EMT markers. (**C**–**D**) Immunofluorescence staining of cells grown for 7 days in 3D BME system for the EMT markers; vimentin and E-Cadherin (E-Cad). (**E**) Western-blot analysis for the sub-cellular expression of LOXL2 in MCF-7-LOXL2 clones. Whole cell extract (WCE), cytoplasmic (Cyto) and nuclear (Nuc) fractionations are presented. Expression of Lamin is used as a control for nuclear fractionations and GAPDH for cytoplasmic fractionations. Magnification ×40, Bar = 50 μm, *n* = 3.

We used two clones of MCF-7 cells stably expressing LOXL2 (MCF-7-LOXL2); Clone #12 [20] and clone #5 (see materials and methods) to test whether they have acquired EMT. MCF-7-LOXL2#12 cells underwent EMT as depicted by loss of the epithelial marker E-Cadherin (E-Cad) and gain of the mesenchymal markers vimentin (Figure 1A). In contrast, MCF-7-LOXL2#5 cells did not acquire an EMT phenotype (Figure 1A). Furthermore, downregulation of LOXL2 expression in MCF-7-LOXL#12 cells by stable expression of sh-LOXL2 (MCF-7-LOXL#12-sh-LOXL2) restored their epithelial phenotype depicted by re-expression of E-Cad. Hence, EMT in MCF-7-LOXL2#12 cells was dependent on LOXL2 expression (Figure 1B). Similarly, MCF-7-LOXL2#12 cells retained their EMT characteristics when cultured in the 3D BME system that models tumor dormancy, depicted by induction of vimentin expression and loss of E-Cad expression (Figure 1C). Conversely, E-cad expression was restored in MCF-7-LOXL2#12-sh-LOXL2 cells cultured in the 3D BME system (Figure 1D). Interestingly, LOXL2 expression in MCF-7-LOXL2#5 cells was mainly confined to the cytoplasm, whereas its expression in MCF-7-LOXL2#12 cells was detected both in the cytoplasm and nucleus (Figure 1E).

**Figure 2:**
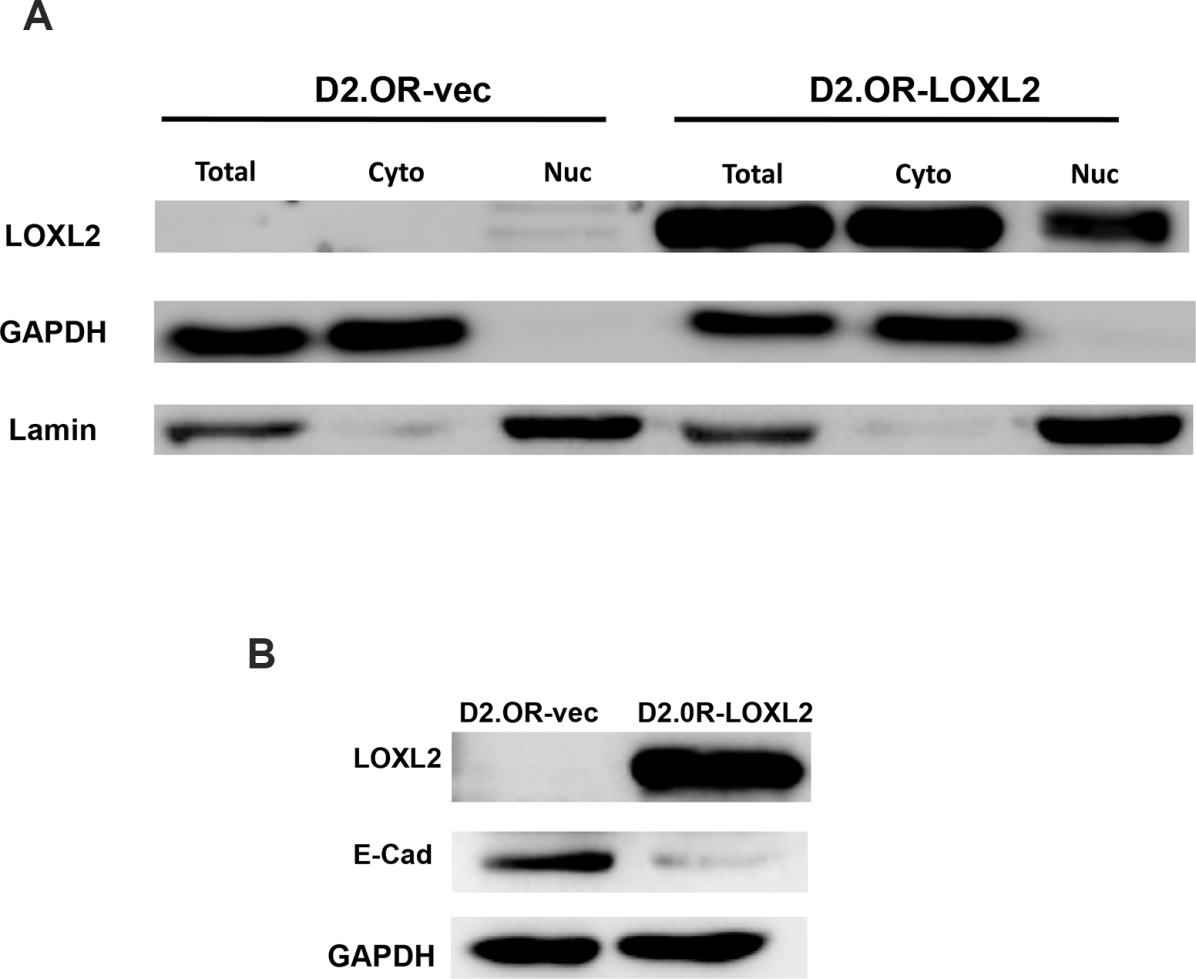
Characterization of D2.0R-LOXL2 cells for LOXL2 sub-cellular localization and E-Cad expression (**A**) Western-blot analysis for the sub-cellular expression of LOXL2 in D2.0R-LOXL2 cells. Whole cell extract (WCE), cytoplasmic (Cyto) and nuclear (Nuc) fractionations are presented. Expression of Lamin is used as a control for nuclear fractionations and GAPDH for cytoplasmic fractionations. (**B**) Western-blot analysis of D2.0R-LOXL2 cells for E-Cad expression.

Similarly, stable expression of LOXL2 in previously described dormant D2.0R mouse mammary cancer cell line [11, 13] was detected both in the cytoplasm and nucleus (Figure 2A) and promoted their EMT depicted by loss of E-Cad expression (Figure 2B). Hence, our results suggest that EMT may be correlated with an increase in nuclear expression of LOXL2 as previously described [26]. Notably, ERα expression was reduced upon LOXL2 expression independent of whether the cells underwent EMT or of the sub-cellular localization of LOXL2 (Figure 1A).

### EMT induced by LOXL2 expression is correlated with the acquisition of a cancer stem-like phenotype

**Figure 3:**
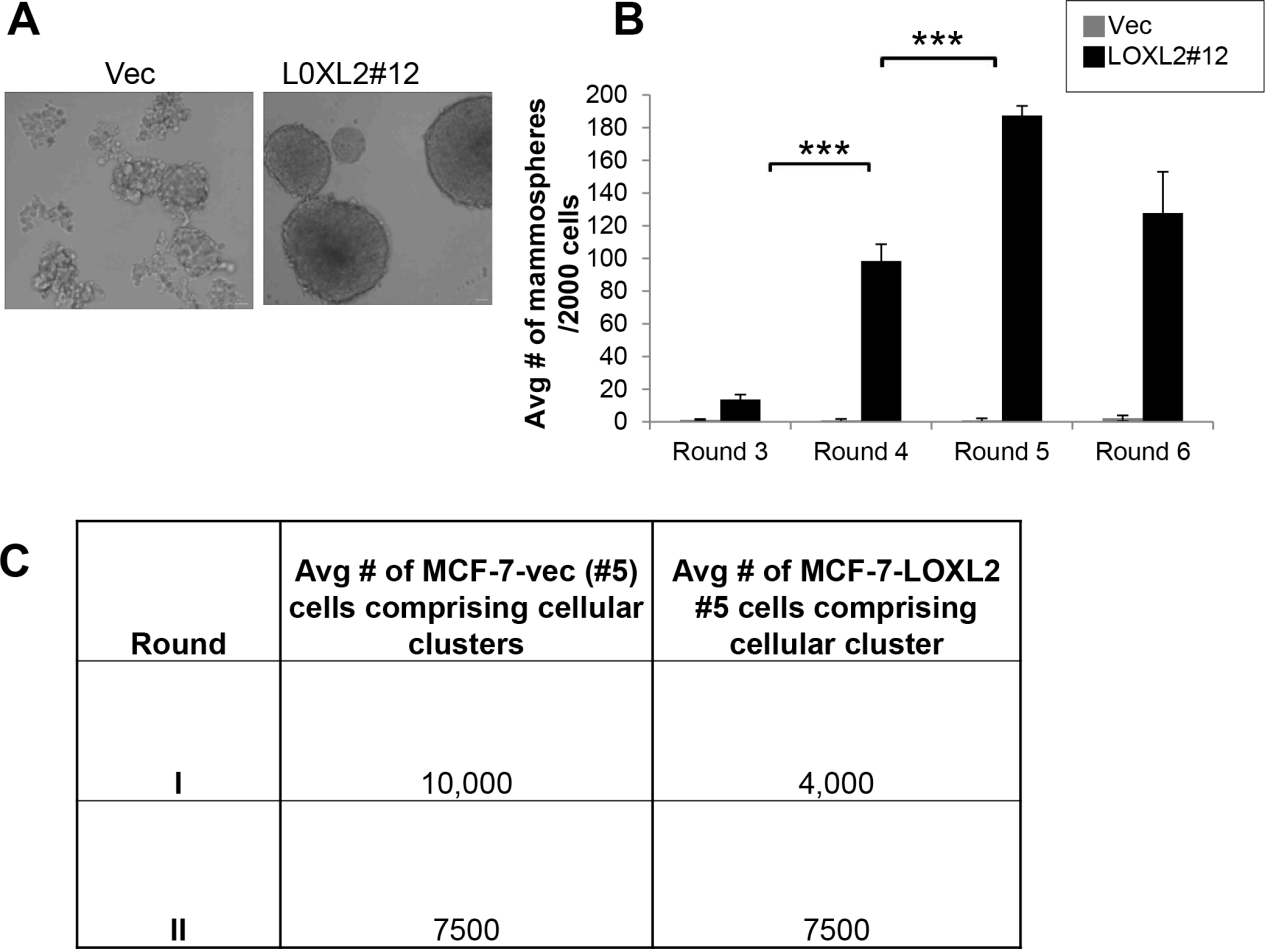
MCF-7-LOXL2 cells with EMT characteristics display high self-renewal capacity (**A**) Light microscopy images of the mammospheres generated by MCF-7-LOXL2#12 cell line (LOXL2#12) compared to control cells (Vec). (**B**) Quantification of the number of mammospheres over 6 generations, each generation 4 replicates. Columns; mean, bars; STD; ***P ≤ 0.001. (**C**) Table representing the average number of cells comprising the first and second generation of cellular clusters generated by MCF-7-LOXL2#5 compared to control cells (Vec).

**Figure 4:**
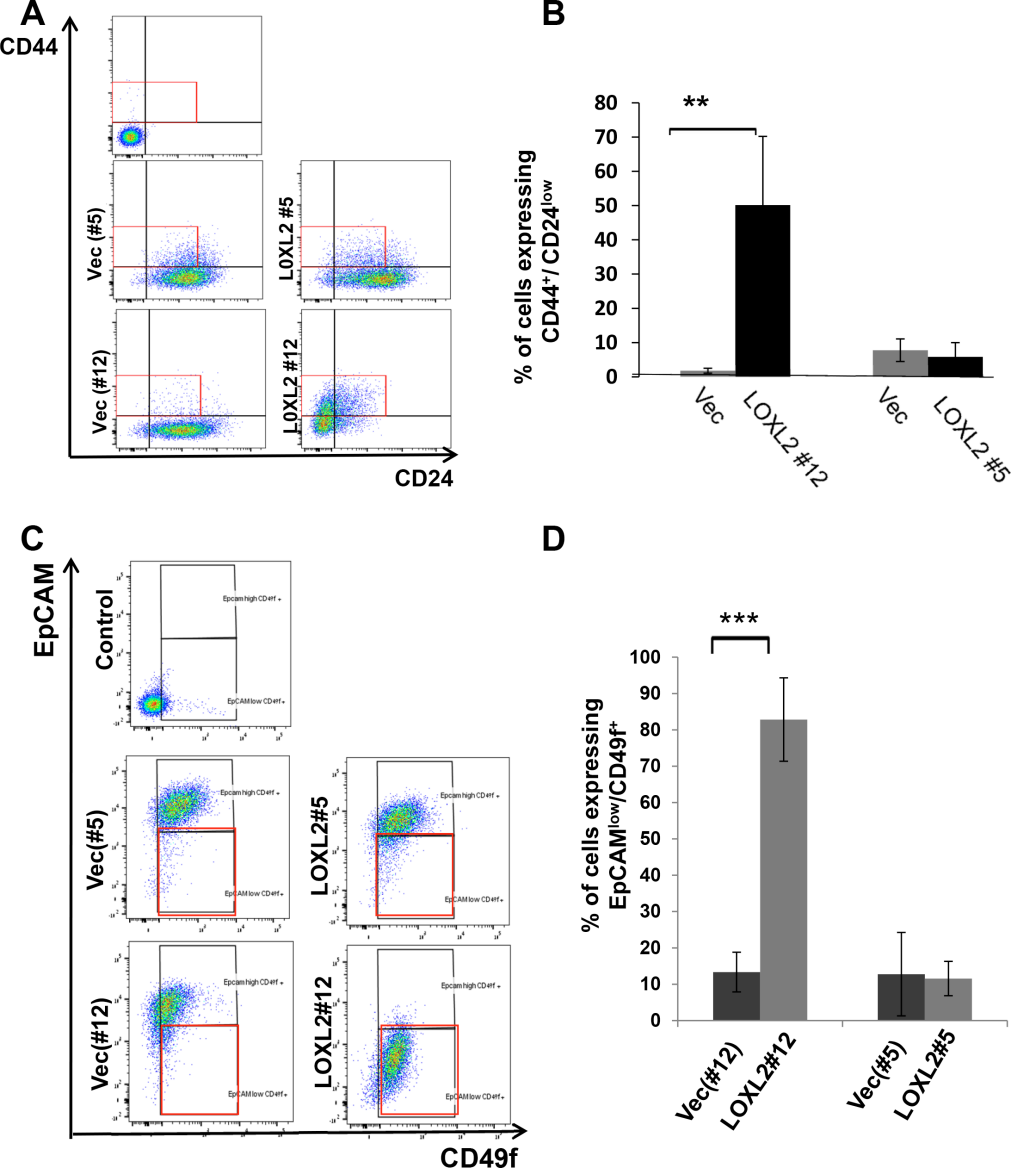
MCF-7-LOXL2 cells with EMT characteristics are enriched with cells expressing CSC markers (**A** and **C**) Representative dot plots of the FACS analysis showing CSC expressing CD44^+^/CD24^low^ (A) or EPCAM^low^/CD49f^+^ (C) phenotype in cells dissociated from secondary mammospheres derived from MCF-7-LOXL2#12 (LOXL2#12) and related control cells (Vec#12) in comparison to MCF-7-LOXL2#5 (LOXL2#5) and related control cells (Vec#5). (**B** and **D**) Quantification of the percentage of CSC. The quantification of the percentage of cells expressing either CD44^+^/CD24^low^ or EPCAM^low^/CD49f^+^ phenotype was carried out with FACSdiva software. Columns; mean, bars; STD; *n* = 3, **P ≤ 0.01, ***P ≤ 0.001.

**Figure 5:**
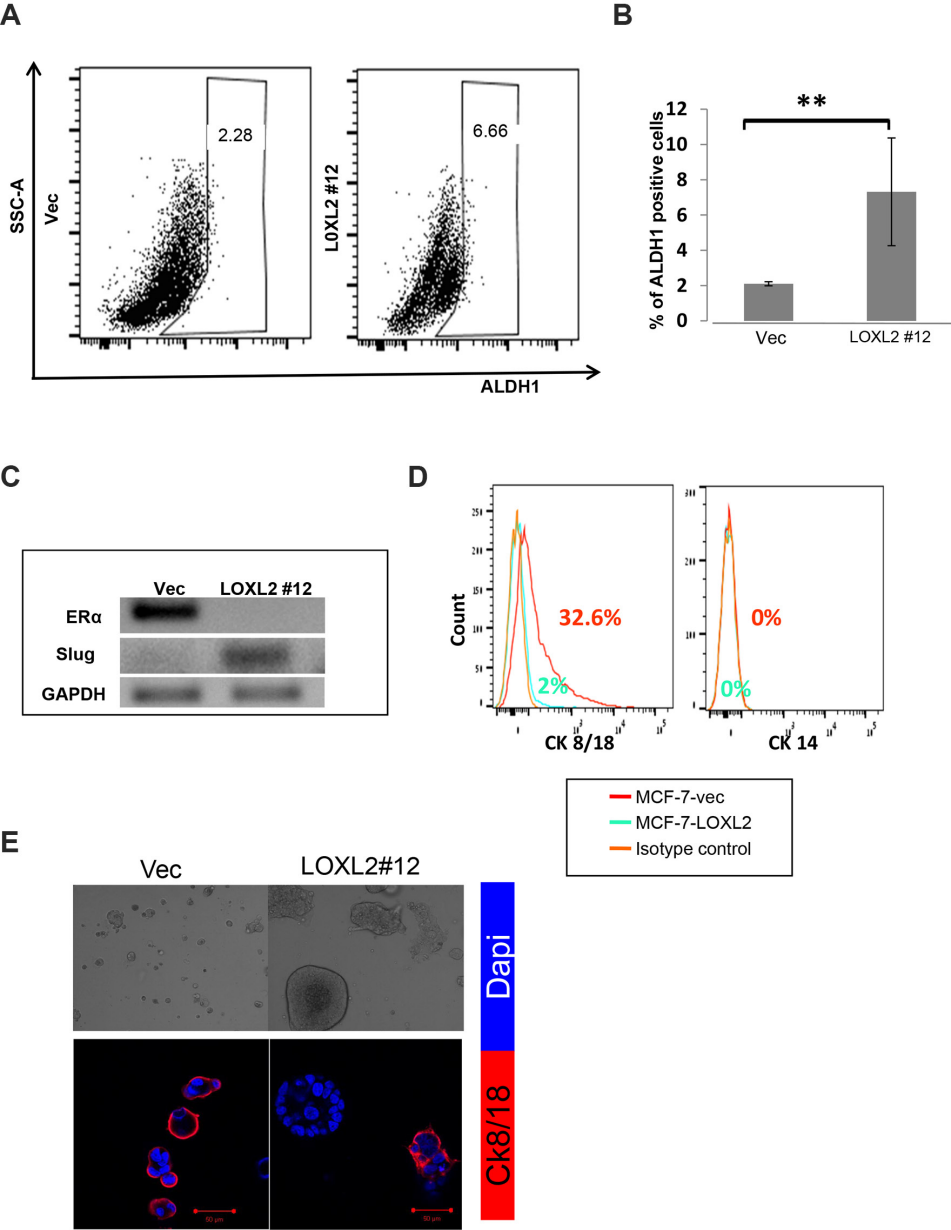
MCF-7-LOXL2 cells with EMT characteristics acquire a CSC-like phenotype (**A**) Representative side scatter plot of the FACS analysis of ALDH1 activity in MCF-7-LOXL2#12 (LOXL2#12) and MCF-7-vec (Vec) cells. (**B**) Quantification of the percentage of ALDH1 positive cells in LOXL2 #12 compared to control (Vec) cells was carried out with FACSdiva software. Columns; mean, bars; STD; *n* = 3, **P ≤ 0.01. (**C**) Semi-qPCR for the expression of Slug and ERα in cells derived from secondary mammospheres. (**D**) FACS analysis for the expression of luminal and myoepithelial lineage markers (Cytokeratin 8/18 and Cytokeratin 14 respectively) in cells dissociated from secondary mammospheres. (**E**) LOXL2 #12 and control Vec cells cultured in the 3D BME system for 7 days. Upper panel: Light microscopy images demonstrating heterogeneity in the morphology of the organoids generated by LOXL2#12 cells. Lower panel: Representative confocal images of immunofluorescence staining for CK/8/18 (Red) and nuclei (Dapi, blue). Magnification ×40, Bar = 50 μm, *n* = 3.

Induction of EMT in transformed human mammary epithelial cells was previously shown to culminate in endowing cells with a stem-like phenotype [27, 28]. Therefore, to test whether MCF-7-LOXL2 cells have potential stem cell-like properties we carried out several *in vitro* assays. A mammosphere assay was carried out to test for self-renewal capacity [29, 30] utilizing MCF-7-LOXL2#12 (LOXL2#12) cells that underwent EMT, MCF-7-LOXL2#5 cells that retained their epithelial phenotype, and their respective control cells (MCF-7-vec). Our results demonstrate that MCF-7-LOXL2#12 cells exhibited a significant increase in their sphere forming capacity for several generations (6 rounds) compared to their control MCF-7-vec cells (Figure 3A–3B). In contrast, MCF-7- LOXL2#5 cells, like their control MCF-vec (#5) cells, did not generate mammospheres and remained either as single cells or formed cell aggregates. Therefore, after the first and second rounds the cells were collected, dissociated, and counted. Indeed, no expansion in cell number was evident in each round of MCF-7- LOXL2#5 cells compared to its control MCF-vec (#5) cells (Figure 3C). Hence, only MCF-7-LOXL2#12 cells display high self-renewal capacity compared to their control cells, suggesting MCF-7-LOXL2#12 cells are enriched with CSC-like cells. We then examined the expression of CSC markers on single cells isolated from second generation of grown mammospheres (secondary mammospheres) by flow cytometry analysis (FACS). The expression of the following CSC markers were evaluated: CD44^+^/CD24^low/−^ and EpCAM^neg/low^/CD49f^high/+^; shown to be expressed by stem cells of normal and cancerous human breast tissue and breast cancer cell lines [30–33] and ALDH1 activity, a surrogate marker for stem cells [34]. MCF-7-LOXL2#12 (LOXL2#12) cells had a significant increase in the percentage of cells expressing CD44^+^/CD24^low/−^ (Figure 4A–4B), EpCAM^low^/CD49f^+^ phenotype (Figure 4C–4D) or ALDH1 activity (Figure 5A–5B) compared to MCF-7-vec cells (MCF-7-vec of #12). Whereas, MCF-7-LOXL2#5 cells (LOXL2#5) had no increase in the subpopulation of cells expressing either CD44^+^/CD24^low/−^ or EpCAM^low^/CD49f^+^ phenotype compared to their control cells (MCF-7-vec of #5) (Figure 4). Thus, EMT promoted by LOXL2 expression is required for acquisition of a CSC-like phenotype.

Notably, the MCF-7-LOXL2#12 mammosphere derived cells expressed the transcription factor Slug and were ERα-negative (Figure 5C). Importantly, Slug and ERα are part of a list of genes characterizing human mammary enriched stem cell population [35]. In addition, an increase in the percentage of undifferentiated cells; depicted by lack of expression of lineage-specific markers (CK14 & CK8/18) characterizing the mammary epithelium, was evident in dissociated cells of secondary mammospheres of MCF-7-LOXL2#12 cells compared to MCF-7-vec cells (Figure 5D). Last, the bi-lineage potential to either differentiate to a basal lineage or luminal lineage (differentiated cell types that comprise the normal breast duct) was evaluated by determining the expression of CK8/18, a mature marker of the luminal lineage, that is absent in mature basal lineage. Indeed, cells derived from the secondary mammospheres of MCF-7-LOXL2#12 cells (enriched for cancer stem cell-like cells) could differentiate to heterogeneous cellular clusters in the 3D BME system with heterogeneous expression (positive and negative expression) of the luminal marker CK8/18 (Figure 5E). Furthermore, the majority of the heterogeneous clusters were negative for CK8/18 (Figure 5E) as expected for a tumor cell culture enriched for CSC-like cells [36].

### DTC acquiring EMT and CSC-like phenotypes will transition from tumor dormancy to proliferative growth in the 3D BME system

**Figure 6:**
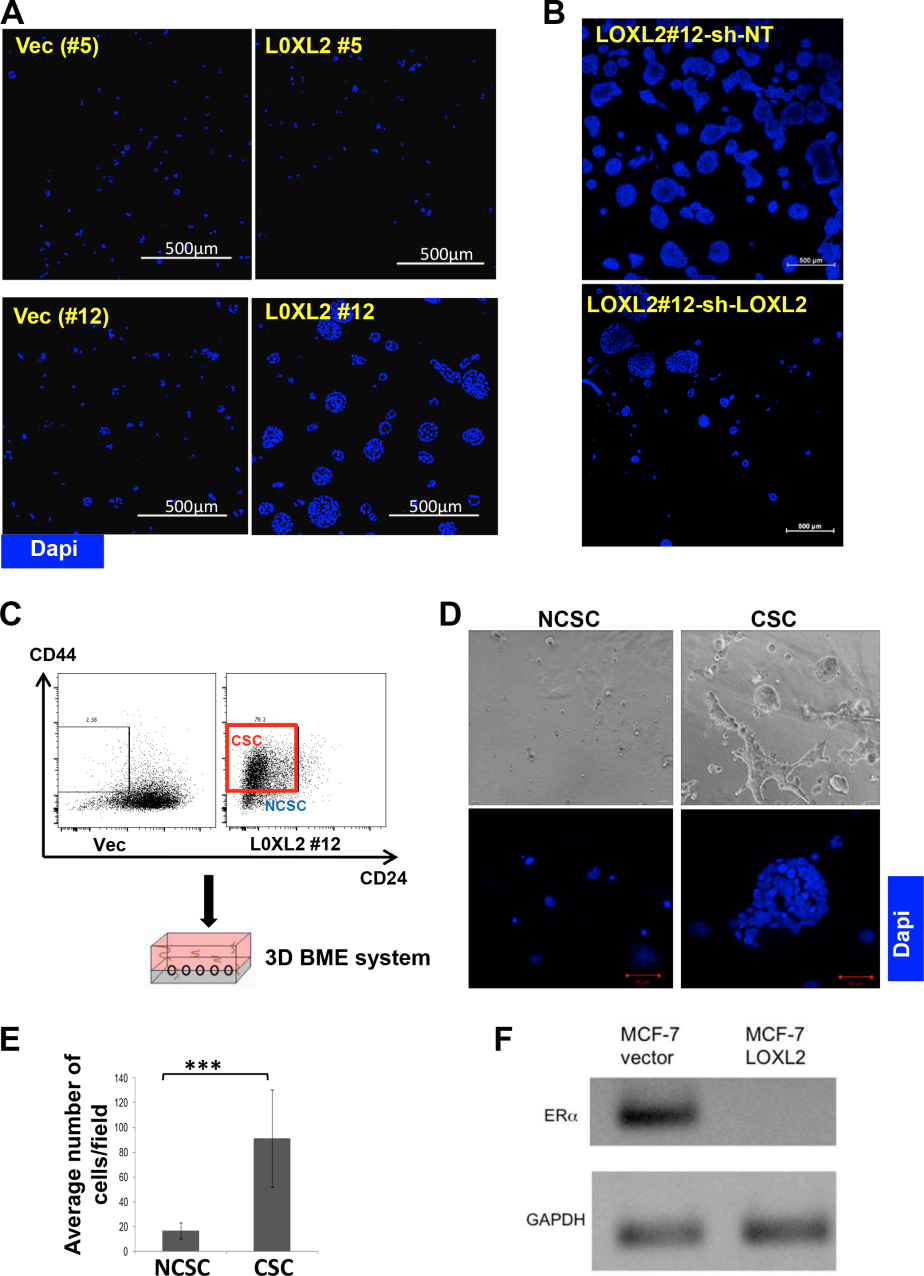
MCF-7-LOXL2 cells with EMT characteristics transition from tumor dormancy to proliferative growth via the CSC population in the 3D BME system (**A**) MCF-7-LOXL2 clones (LOXL2#12, LOXL2#5) and their related control cells (Vec #12 and Vec #5 cells respectively) and (**B**) MCF-7-LOXL2 #12 cells stably expressing either sh-non-target (sh-NT) or sh-LOXL2 (sh-LOXL2) were cultured in the 3D BME system for 3 days (A) and for 7 days (B) and stained for nuclei (Dapi, blue). Confocal image of 5 × 5 fields (A) and 10 × 10 fields (B) are presented, magnification ×40; Bars=500 μm. (**C**) Scheme demonstrating the sorting of CSC expressing CD44^+^ CD24^low/−^ phenotype (red square) and the rest non-CSC (NCSC) from secondary mammospheres of LOXL2#12 cells, and their culture in the 3D BME system. (**D**) Flow sorted CSC and NCSC cultured in the 3D BME system for 7 days. Upper panel: Representative Light microscopy images. Bottom panel: Representative confocal images of the cells stained for nuclei (Dapi, blue). Magnification ×40, Bar = 50 μm. (**E**) Quantification of the number of CSC and NCSC cells in 3D BME culture. Average number of cells/12 fields was scored for NCSC and average number of cells/4 fields was scored for CSC. The quantification of the number of cells /fields was carried out with Imaris software. Columns; mean, bars; STD; *n* = 3, ***P ≤ 0.001. (**F**) Semi-qPCR for the expression of ERα in LOXL2#12 and control Vec cells cultured in the 3D BME system.

The 3D BME culture system was used as a model for DTC as previously described [13, 25]. We have previously shown that MCF-7 cells exhibit dormant (quiescent) behavior when cultured in the 3D BME system, correlative with its behavior at distant sites *in-vivo* [13]. Here we demonstrate that MCF-7-LOXL2#12 cells transition from quiescence to proliferation in the 3D BME system, whereas control cells or MCF-7-LOXL2#5 cells that didn't undergo EMT remained dormant (Figure 6A). Furthermore, downregulation of LOXL2 expression in MCF-7-LOXL2#12 cells (MCF-7-LOXL2#12-shLOXL2) inhibited their outgrowth (Figure 6B). These results suggest that DTC that express LOXL2 and undergo EMT will transition to metastatic outgrowth. Moreover, flow sorted CSC (cells expressing CD44^+^/CD24^low/−^ phenotype; [27, 37]) from secondary mammospheres of MCF-7-LOXL2#12 cells (Figure 6C) transitioned from quiescence to proliferative growth when cultured in the 3D BME system (Figure 6D–6E). Whereas, flow sorted non-cancer stem cells (NCSC) (the rest of the sorted cells; Figure 6C) remained dormant in the 3D BME system (Figure 6D–6E). Notably, the outbreaking MCF-7-LOXL2#12 cells no longer expressed ERα (Figure 6F). All together, our results demonstrate that EMT induced by LOXL2 expression in dormant MCF-7 cells promoted their emergence from tumor dormancy to proliferative growth and the CSC-like population mediated this transition.

### LOXL2 expression in MCF-7 cells promotes their escape from tumor dormancy *in vivo*

**Figure 7:**
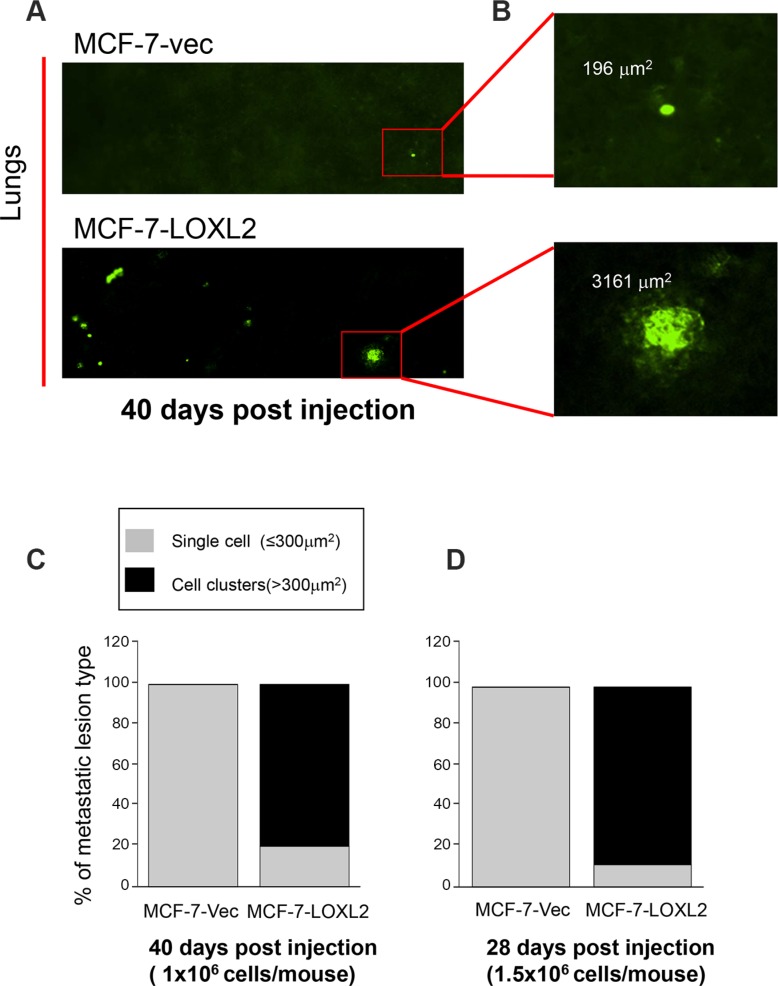
LOXL2 expression in dormant MCF-7 cells promotes their transition from tumor dormancy to metastatic growth *in vivo* (**A**) SCOM images of MCF-7-vec-GFP (MCF-7-vec) and MCF-7-LOXL2#12-GFP (MCF-7-LOXL2) lung lesions in mice. Magnification ×10. (**B**) Digital zooming of the selected area. Upper panel: single cells (foci ≤ 300 mm^2^). Lower panel: multi-cellular proliferative metastatic lung lesions (cell clusters > 300 mm^2^). (**C**–**D**) Percentage of dormant single cells versus multi-cellular proliferative metastatic lung lesions in mice as depicted in panel B. (C) 1 × 10^6^ cells/mouse were injected and lungs were harvested and imaged by SCOM 40 days post injection, *n* = 3; *p* ≤ 0.01 across all samples. (D) 1.5 × 10^6^ cells/mouse were injected and lungs were harvested and imaged by SCOM 28 days post injection, *n* = 3; *p* ≤ 0.001 across all samples.

We performed experimental metastasis assay by tail vein injection of nude mice either with MCF-7-vec-GFP or MCF-7-LOXL2#12-GFP cells that were also labeled with a cell-tracker. Initially, we injected 1 × 10^6^ cells/mouse and harvested the lungs after 40 days. Tumor cells on the surface of the lungs were imaged by single cell organ microscopy (SCOM) (Figure 7) and multi-cellular metastatic lesions and individual metastatic cells expressing GFP were quantified on the entire lung surface. Fluorescence surface area of > 300 mm^2^ represented multi-cellular, proliferative metastatic lesions, whereas foci of ≤ 300 mm^2^ indicated individual, dormant metastatic cells (Figure 7A–7B). All metastatic lesions developed by control MCF-7-vec-GFP cells persisted as single quiescent cells (100%) (Figure 7C). Whereas, in mice that were injected with MCF-7-LOXL2#12-GFP cells only 20% of the lung lesions remained as single tumor cells and 80% formed multi-cellular lesions (*P* ≤ 0.01) (Figure 7C). We next tested whether injecting a higher number of cells (1.5 × 10^6^ cells/mouse) will expedite the outgrowth of the dormant tumor cells. Indeed after 28 days 89% of the lung lesions of MCF-7-LOXL2#12-GFP cells formed multi-cellular lesions, in contrast all metastatic lesions developed by control MCF-7-vec-GFP cells persisted as single quiescent cells (100%) (*P* ≤ 0.001) (Figure 7D). All together our results demonstrate that dormant MCF-7 cells will escape tumor dormancy upon LOXL2 expression.

### Hypoxia induced EMT and CSC-like phenotype in MCF-7 cells is mediated by LOXL2 expression

**Figure 8:**
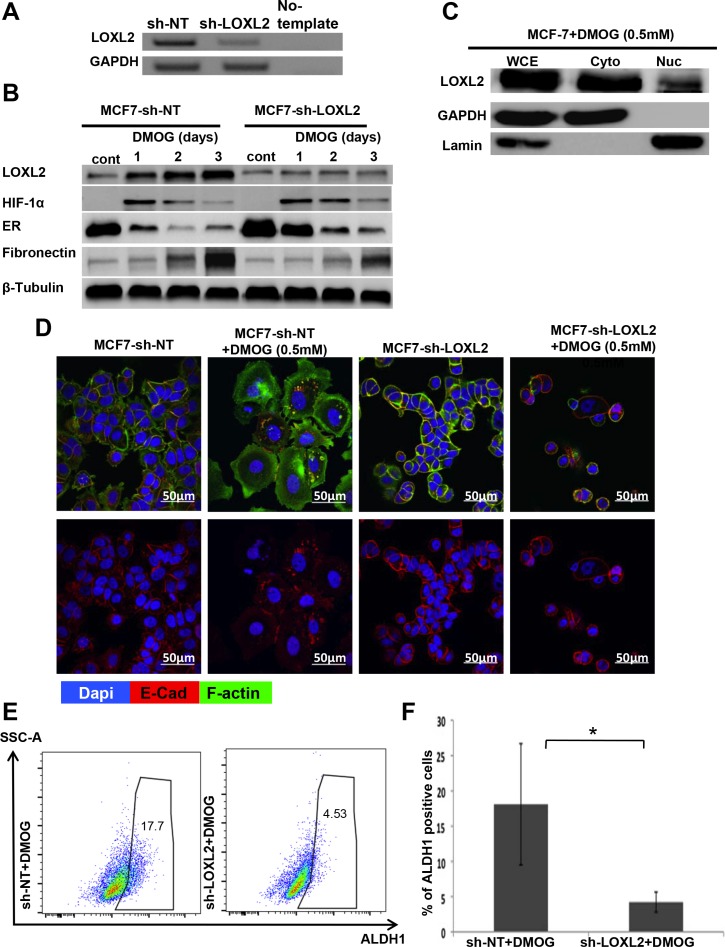
LOXL2 mediates hypoxia induces EMT and CSC-like phenotype of MCF-7 cells (**A**) Semi-qPCR analysis for the knockdown of LOXL2 expression in MCF-7 cells. (**B**) MCF-7-sh-non-target and MCF-7-sh-LOXL2 cells treated with DMOG (0.5 mM). Cells were sampled over 3 days of exposure to DMOG and analyzed by western blot analysis for the expression of LOXL2, HIF-1α, EMT marker fibronectin and luminal marker ERα. (**C**) Western-blot analysis for the sub-cellular expression of LOXL2 in MCF-7 cells treated with DMOG (0.5 mM) for 2 days. Whole cell extract (WCE), cytoplasmic (Cyto) and nuclear (Nuc) fractionations are presented. (**D**) Immunofluorescence staining of MCF-7-sh-non-target and MCF-7-sh-LOXL2 cells either untreated or treated with DMOG (0.5 mM) for 3 days for E-Cad (Red), F-actin (green) and nuclei (Dapi, blue). Representative confocal images, magnification ×40, Bars = 50 μm. (**E**) Representative side scatter plot of the FACS analysis of ALDH1 activity in MCF-7-sh-non-target and MCF-7-sh-LOXL2 cells treated for 3 days with DMOG and quantification of the percentage of ALDH1 positive cells (**F**). Columns; mean, bars; STD; *n* = 3, *P ≤ 0.05

Hypoxia is part of the tumor microenvironmental milieu. Here we tested whether endogenous LOXL2 expression in MCF-7 cells will be induced by promoting the stabilization of the hypoxia inducible factor-1α (HIF-1α), resulting in EMT (expression of fibronectin and cytoplasmic localization of E-Cad) and thus endowing the cells with stem-like properties. We utilized MCF-7 cells either stably expressing sh-non-target or sh-LOXL2 (Figure 8A). Our results demonstrate that stabilizing HIF-1α expression in MCF-7-sh-non-target cells, by treating the cells with dimethyloxalylglycine (DMOG; 0.5 mM) induced LOXL2 expression in contrast to no induction of LOXL-2 expression in MCF-7-sh-LOXL2 cells (Figure 8B). Furthermore, DMOG induced both cytoplasmic and nuclear expression of LOXL2 (Figure 8C). Notably, silencing LOXL2 expression in MCF-7 cells inhibited hypoxia induced EMT as depicted by inhibition of mesenchymal phenotype (see F-actin organization; Figure 8D), inhibition of fibronectin expression (Figure 8B) and membrane localization of E-Cad (Figure 8D).

Furthermore, silencing LOXL2 expression in DMOG treated MCF-7 cells significantly inhibited the percentage of CSC-like cells (Figure 8E–8F) and delayed the reduction in ERα expression (Figure 8B). Altogether, our data demonstrates that microenvironmental cues such as hypoxia can induce endogenous LOXL-2 expression in MCF-7 cells, promoting EMT and CSC-like phenotype.

### Decrease in relapse free survival of breast cancer patients expressing high levels of LOXL2 and EMT/CSC markers

**Figure 9:**
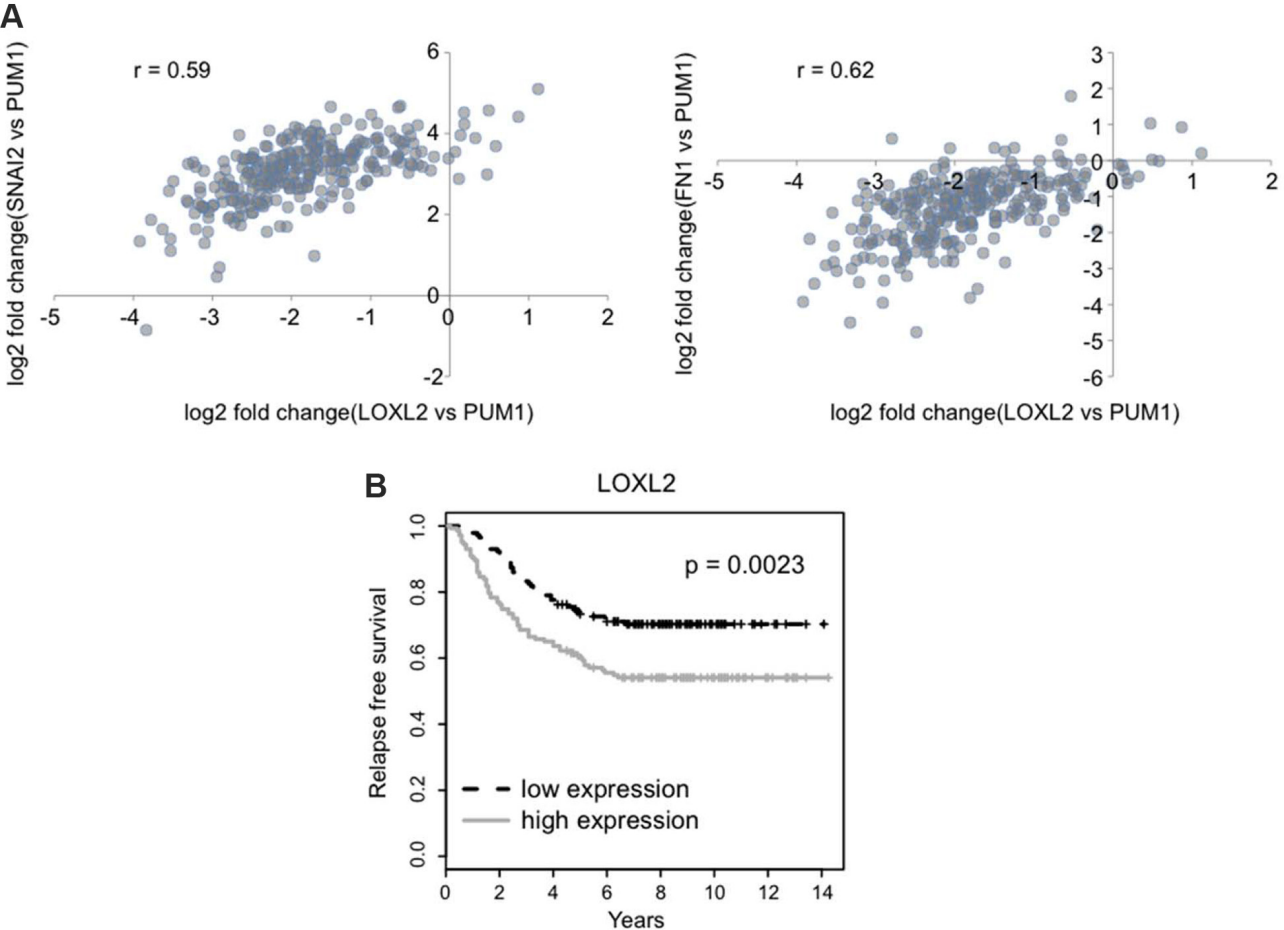
LOXL2 expression correlates with EMT/CSC markers and is associated with decreased relapse free survival (**A**) Expression levels of the *LOXL2*, *SNAI2* (Slug) and *FN1* (fibronectin) genes were normalized to the expression of the *PUM1* gene, which is stably expressed across various breast cancer tissues (REFERENCE PMID: 23720140). Scatter plots show the correlation between expressions of given genes (r – Pearson's correlation coefficient). (**B**) Relapse free survival of two groups is shown in Kaplan-Meier plot (*p*-value for the log-rank test).

The clinical implications of our findings were further assessed by the analysis of GSE2034 breast cancer relapse free survival data set (REFERENCE PMID: 15721472). This series represents 180 lymph-node negative relapse free patients and 106 lymph-node negative patients that developed a distant metastasis. Our results demonstrate that increased LOXL2 mRNA levels correlate with increase in the mRNA levels of the EMT markers such as fibronectin (FN1) and Slug (SNAI2) [38], the latter was previously found to be also one of the surrogate markers of human mammary stem cells [35] (Figure 9A). Moreover, we demonstrate that increase in mRNA levels of LOXL2 (Figure 9B) is associated with significant decrease in the relapse free survival (RFS) in patients with lymph node-negative breast cancers. Hence, these findings demonstrate that breast cancer patients with increased LOXL2 expression have a higher risk to develop recurrence of the disease, and increase in LOXL2 expression is associated with increase in EMT/CSC like markers.

## DISCUSSION

The mechanisms underlying the emergence of DTC to metastatic growth is largely unknown. Here we demonstrate that LOXL2, beyond its extracellular function as inducer of desmoplastic stroma [14, 20] and its intracellular function as an inducer of EMT and cancer cell invasion [18, 21, 26], can also endow DTC with CSC–like properties thus mediating their emergence from dormancy to proliferative growth. We demonstrated that dormant D2.0R and MCF-7 cells expressing both cytoplasmic and nuclear LOXL2 undergo EMT (as was previously shown for MCF-7 cells [26]). This pattern of LOXL2 expression was previously reported to be present in basal breast tumors and was strongly correlated with distant metastasis incidence [18]. Notably, nuclear LOXL2 was previously shown to interact and cooperate with E-Cad repressor Snail [39] and with the bHLH transcription factor E47 to downregulate E-Cad expression [24], a hallmark of EMT. Our results demonstrate that MCF-7-LOXL2 cells that underwent EMT acquired CSC-like properties. CSC-like properties were evaluated by several *in vitro* assays designed to determine their capacity for self-renewal, as well as the level of expression of CSC markers such as CD44^+^/CD24^low/−^, EpCAM^low^/CD49f^+^ and ALDH1 activity. The undifferentiated status is one of the hallmarks of stem cells [30] and here we have demonstrated that vast majority of the cell isolated from second generation of grown mammospheres of MCF-7-LOXL2 cells that underwent EMT, lacked the expression of lineage-specific markers of the mammary epithelium such as CK14 and CK8/18. Furthermore, these cells expressed the transcription factor Slug, previously found to be one of the surrogate markers of human mammary stem cells [35] and was reported to regulate the maintenance of human breast CSC [40]. Intriguingly, MCF-7-LOXL2 cells enriched with CSC population displayed a bi-lineage potential when cultured in the 3D BME system, as revealed by the heterogeneous expression of the luminal linage marker CK-8/18. Hence, the multi-lineage potency further demonstrates that these cells have acquired stem-like traits. Interestingly, it was recently demonstrated that LOXL2 plays a role in maintaining the balance between the pluripotency and differentiation of embryonic stem cells to neural progenitors [41], further supporting the novel role of LOXL2 in modulating the stem and or progenitor cell traits.

All together our results suggest that EMT of DTC can endow the cells with CSC-Like traits. These finding are in concordance with previous studies demonstrating the role of EMT in endowing cells with CSC-like traits [27, 28]. Here we show that EMT induced by LOXL2 expression in DTC promoted their transition from tumor dormancy to proliferative growth in the 3D BME system, whereas cells that retained their epithelial phenotype remained dormant. These findings are in concordance with a previous study demonstrating the role of EMT in the switch from tumor dormancy to proliferative growth [42]. Furthermore, we demonstrated that induction of EMT following LOXL2 expression endows the cells with CSC-like properties. Intriguingly, only the subpopulation of cells with CSC-like properties mediated the outgrowth of the dormant tumor cells, whereas the rest of the cells expressing LOXL2, which do not display CSC-like properties, remained dormant in the 3D BME system. Likewise, we demonstrated *in vivo* that MCF-7-LOXL2-GFP cells enriched with CSC-like cells escaped tumor dormancy, whereas control MCF-7-vec-GFP cells remained dormant 28 and 40 days post injection as was previously shown [13]. Similarly, Malanchi and colleagues previously demonstrated that only the CSC population was capable to initiate metastatic nodules at secondary site [43]. Notably, our findings demonstrate that the tumor microenvironment can promote DTC to acquire CSC-like phenotype by inducing LOXL2 expression, thus mediating their transition to metastatic outgrowth. Specifically hypoxia, which is part of the tumor microenvironment milieu, previously shown to play an important role in establishing pre-metastatic niche [44], can induce endogens expression of cytoplasmic and nuclear expression of LOXL2 resulting in EMT and a CSC-like phenotype of MCF-7 cells. In contrast, silencing LOXL2 expression in MCF-7 cells prevented hypoxia induced EMT and prevented the increase in CSC-like cells as depicted by ALDH1 activity.

Taken together our study demonstrates that the tumor microenvironment can promote DTC to acquire a CSC-like phenotype via EMT mediated by LOXL2 expression resulting in their outgrowth and loss of their luminal phenotype.

Interestingly, analysis of breast cancer relapse free survival data set revealed that increase in LOXL2 expression is correlated with increase in the EMT/CSC markers fibronectin and Slug. Furthermore, increase in LOXL2 expression was associated with significant decrease in RFS of the breast cancer patients. Overall, our results suggest that determining LOXL2 expression levels may serve as a good prognostic marker for the RFS of breast cancer patients. This may pave the way for new therapeutic strategies targeting cellular LOXL2 in addition to extracellular LOXL2, as means to prevent and treat metastatic recurrence of breast cancer disease.

## MATERIALS AND METHODS

### Reagents cell culture transfections and viral infections

Cells were grown in Dulbecco's modified Eagle's medium (DMEM) supplemented with 4.5 g/ml D-glucose containing 10% fetal bovine serum and 1% penicillin and streptomycin, in a 5% CO2 incubator. MCF-7 cells were purchased from American Type Culture Collection (ATCC) and were stably transected with pCDNA3-LOXL2 [20] using ESCORT transfection reagent (Sigma–Aldrich Ltd.) and selected by puromycin (Gold Biotechnology). MCF-7-shLOXL2 cells were prepared as previously described [45]. D2.0R-LOXL2 cells were prepared by lentiviral infection of D2.0R cells using the NSPI-CMV-Myc lentiviral expression vector for human LOXL2 [46] and selection was performed by 2 μg/ml puromycin.

Three-dimensional cultures were carried out as previously described [13, 25]. Briefly, eight-chamber glass slides (Lab -TEK^®^ II, Naperville, IL) were coated with 60 μl of growth factor reduced BME Cultrex^®^ (BME) (Trevigen Inc., Gaithersburg, MD). 5 × 10^3^ cells/well were resuspended in DMEM low glucose + 2% FBS supplemented with 2% BME.

### Mammosphere assay

Mammosphere assay was carried out as previously described [30]. Briefly, 10 × 10^3^ cells/ml were cultured in low attachment surface plates (bacterial plates) for mammosphere enrichment (Corning) or 2 × 10^3^ cells were cultured in 24 well ultra-low attachment surface plates (Greiner bio-one) for self-renewal assay. Cells were cultured in mammosphere medium (DMEM/F12 medium supplemented with B27 (Gibco life technology), EGF and bFGF- 20 ng/ml, Heparin- 2 μg/ml (Peprotech) and pen-strep (Biological Industries)).

Progression for several generations: Spheres were collected at day 7 and centrifuged (1500 g for 5 minutes). Pellets were re-suspended with 1 ml of trypsin followed by 5 min incubation. Spheres were disaggregated using syringe with 21G needle, to obtain single cell suspension, counted, and cultured as described above according to the assay to be conducted; either for CSC enrichment or self renewal assay. Quantification of the number of mammospheres for the self-renewal assay was done by light microscopy at magnification ×10 for counting all mammospheres /field. Experiments were conducted with 4 replicates for each round.

### Western blot

Western blot was carried out as previously described [13]. The following primary antibodies were used (See also Supplementary Table): anti LOXL2 [20] 1:10,000, ER, GAPDH, Lamin, and β-Tubulin (1:500; Santa-Cruz). Anti-E-Cadherin (1:500), Vimentin (1:2000), HIF-1α (1:2000) and Fibronectin (1:1000) (abcam). Horseredish peroxidase-conjugated secondary antibodies (1:10,000; Jackson ImmunoResearch Laboratories).

### Nuclear/Cytoplasmic fractionation

Nuclear/Cytoplasmic fractionation assay was carried out as previously described [47]. Cells were washed twice with PBS, scraped and collected on ice into 1.5 ml microcentrifuge tubes. Tubes were spun with table-top centrifuge, and supernatant was discarded. Fractionation was preformed with 0.1% NP40-PBS treatment:

Cell pellets were triturated 5 times with ice-cold 0.1% NP40-PBS (900 μl for 10 cm dish) using p1000 micropipette that was cut at its end. Aliquots of 300 μl of these samples were placed into fresh tubes (designated as Total). The remaining samples were centrifuged for 1 min 16,200 g to pellet nuclei. Aliquots of 300 μl of the supernatant were collected into fresh tubes (designated as Cyto). 100 μl of 4 × Laemmli sample buffer was added immediately to Total and Cyto samples.

Nuclei pellets were resuspended with ice-cold 0.1% NP40-PBS (1 ml for 10 cm dish), re-pelleted, and resuspended with 180 μl of 1 × Laemmli sample buffer (designated as Nuc).

Finally, Total and Cyto samples were sonicated using microprobes at level 2, twice for 5 sec. Aliquots of DMOG treated fractionation samples were collected for protein determination, before the addition of Laemmli sample buffer.

### Flow-cytometric analysis (FACS)

Dissociated single cells from secondary mammopsheres were characterized for CSC properties by staining the cells with: 1) anti CD44-FITC and anti CD24-APC (Bio-legend) antibodies. We defined CD24^low^ gating based on MFI that was ×10 fold lower then the MFI of CD24^high^. 2) Monoclonal antibodies against-Cytokeratin 8/18 and Cytokeratin 14 (ABD serotec) following permeabilization of the cells with Leucoperm (ABD serotec). PerCp conjugated anti mouse secondary antibody was used (Jackson ImmunoResearch) and 3) ALDH1 activity using ALDEFLUOR^TM^ kit (STEMCELL technologies) according to the manufacturer protocol. Gating was determined as 1% ALDH1 activity in the inhibitor reference test. Dead cells were eliminated by forward and side scatter analysis. All FACS analysis was done using FACSCanto II (BD). Sorting was performed using FACS Aria–IIIu Sorter (BD).

### Immunofluorescence staining

Immunofluorescence staining was carried out as previously described [25]. Briefly, cells were cultured in 8 well chamber glass slides, fixed for 5 min with 4% PFA containing 5% sucrose and 0.1% Triton X-100, and re-fixed for an additional 25 min with 4% PFA containing 5% sucrose. The cells were washed 10 min with PBS and an additional 10 min with PBS containing 0.05% Tween 20. Fixed cells were blocked with IF buffer (130 mM NaCl, 7 mM Na_2_HPO_4_, 3.5 mM NaH_2_PO_4_, 7.7 mM NaN_3_, 0.1% BSA, 0.2% Triton X-100, 0.05% Tween 20) supplemented with 10% donkey serum for 1 hour and incubated overnight at 4°C with primary antibody (dilution according to Supplementary Table S1). The cells were washed three times with PBS for 15 minutes each, and incubated for 1 hour with donkey anti–respective-IgG conjugated to either Alexa Fluor^®^647 or Alexa Fluor^®^568 (Invitrogen) (dilution according to Supplementary Table S1), washed as above, and mounted with VECTASHIELD mounting medium with 4′, 6-diamidino-2-phenylindole (DAPI). For F-actin staining, cells were incubated overnight with Alexa-Fluor^®^488 Phalloidin (1:40) (Molecular Probes), washed three times with PBS for 15 minutes each, and mounted with VECTASHIELD mounting medium with DAPI. Immunofluorescent images were captured either by Zeiss LSM 700 confocal laser scanning microscope or Nikon A1R confocal microscope.

### Semi-quantitative RT-PCR

RNA was reversed-transcribed using the High Capacity RNA-to-cDNA Kit (Applied Biosystems). The cDNA was used as a template for semi-quantitative PCR using the PCR Dream-Taq Mix (Thermo-scientific).

Human Slug primers: F-5′-ATGAGGAATCT GGCTGCTGT-3′, R- 5′CAGGAGAAAATGCCTTTGGA-3′; human ER primers: F-5′-AAGAGCTGCCAGGCCT GCC-3′, R-5′-TTGGCAGCTCTCATGTCTCC-3′; human GAPDH primers: F-5′- ATGGGGAAGGTGAAGGTCG-3′, R-5′- GGGGTCATTGATGGCAACAATA-3′,

human LOXL2 primers: F-5′- ACATGTACCGC CATGACATCGACT-3′, R-5′-TGAAGGAACCACCTAT GTGGCAGT-3′.

### Experimental metastasis assays

6–8 week-old female BALB/c-nu/nu athymic mice were injected via tail vein with 1–1.5 × 10^6^ cells stably expressing GFP labeled with cell tracker CSFE (Sigma) according to the manufacture protocol. Lungs were removed 28 and 40 days post injection, inflated with PBS and subjected to fluorescent single cell whole organ microscopy (SCOM) imaging by fluorescent video-microscopy (Nikon A1R Microscopy) as previously described [11, 13]. 10 X images of the entire external surface of each lung were sequentially captured and analyzed using NIS-Elements AR software to measure the surface area of the metastases. Surface area > 300 mm^2^ represented multi-cellular, proliferative metastatic lesions, whereas 60 mm^2^ > foci ≤ 300 mm^2^ indicated individual, dormant metastatic cells. All mice were maintained under specific pathogen-free conditions. Care and handling of animals was in compliance with Technion-Israel Institute of Technology experimental protocols.

### Relapse free survival calculation

A previous study (REFERENCE PMID: 15721472) has provided expression levels of whole transcripts in 286 lymph-node negative patients with their clinical information. The data deposited in gene expression omnibus (GSE2034) was downloaded to calculate relapse free survival by means of Kaplan-Meier (K-M) estimates. Briefly, patients were categorized into two groups according to expression level of the *LOXL2* gene (top 50%: high expression; the rest: low expression). Kaplan-Meier plot for relapse free survival of two groups was drawn using the survival package provided in the R software (https://www.r-project.org/). Log-rank test was conducted to calculate *p*-value.

### Statistical analysis of *in vitro* and *in vivo* experiments

Student's unpaired *t* test was used for data analysis. Two tailed *p* values of 0.05 or less were considered to be statistically significant.

## SUPPLEMENTARY MATERIALS TABLE


