# Supplementary material for: Dormant tumor cells expressing LOXL2 acquire a stem-like phenotype mediating their transition to proliferative growth
Source: Oncotarget. 2016 Sep 19;7(44):71362–77. doi: 10.18632/oncotarget.12109 (PMC5342084; doi:10.18632/oncotarget.12109)
Supplement: Supplementary file 1 [file oncotarget-07-71362-s001.pdf]

Dormant tumor cells expressing LOXL2 acquire a stem-like phenotype mediating their transition to proliferative growth

Supplementary Materials

Supplementary Table S1: List of antibodies

Application	Antibody	Company	Cat#	Dilution
**W.B**	LOXL2	Self-production by Gera Neufeld		×10000
	ER	santa-cruz	sc-542	×500
	E-Cad (human)	abcam	ab1416	×500
	E-Cad (mouse)	abcam	ab76055	×1000
	Vimentin	abcam	ab92547	×2000
	GAPDH	santa-cruz	sc25778	×500
	Lamin	santa-cruz	sc6217	×100
	HIF1α	abcam	ab16066	×2000
	Fibronectin	abcam	ab2413	×1000
	β-Tubulin	santa-cruz	sc9104	×500
**IF**	Vimentin	abcam	ab92547	×200
	E-Cad (human)	abcam	ab1416	×50
	AF 647 Donkey α Rabbit	Molecular probes	A31573	×200
	AF 568 Donkey α mouse	Molecular probes	A10037	×200
	CK 8-18	ab17139	ab17139	×100
**FACS**	FITC α CD44	Biolegend	338804	5 µL/million cells
	APC α human CD24	Biolegend	311118	5 µL/million cells
	CK 8-18	abcam	ab17139	4 µL/million cells
	CK 14	BD		2 µL/million cells
	APC α EPCAM (CD326)	Biolegend	324208	5 µL/million cells
	AF488 CD49f	Biolegend	313608	5 µL/million cells
